# Effects of low-flux and high-flux hemodialysis on the survival of elderly maintenance hemodialysis patients

**DOI:** 10.1080/0886022X.2024.2338217

**Published:** 2024-04-07

**Authors:** Wanqing Huang, Jiuxu Bai, Yanping Zhang, Dongxia Qiu, Lin Wei, Chen Zhao, Zhuo Ren, Qian Wang, Kaiming Ren, Ning Cao

**Affiliations:** a Postgraduate., Training Base of Jinzhou Medical University (General Hospital of Northern Theater Command), Jinzhou, China; b Department of Blood Purification, General Hospital of Northern Theater Command, Shenyang, China

**Keywords:** High-flux, low-flux, hemodialysis, elderly, survival

## Abstract

**Background:**

Elderly hemodialysis (HD) patients have a high risk of death. The effect of different types of HD membranes on survival is still controversial. The purpose of this study was to determine the relationship between the use of low-flux or high-flux membranes and all-cause and cardiovascular mortality in elderly hemodialysis patients.

**Methods:**

This was a retrospective clinical study involving maintenance hemodialysis patients which were categorized into low-flux and high-flux groups according to the dialyzer they were using. Propensity score matching was used to balance the baseline data of the two groups. Survival rates were compared between the two groups, and the risk factors for death were analyzed by multivariate Cox regression.

**Results:**

Kaplan–Meier survival analysis revealed no significant difference in all-cause mortality between the low-flux group and the high-flux group (log-rank test, *p* = 0.559). Cardiovascular mortality was significantly greater in the low-flux group than in the high-flux group (log-rank test, *p* = 0.049). After adjustment through three different multivariate models, we detected no significant difference in all-cause mortality. Patients in the high-flux group had a lower risk of cardiovascular death than did those in the low-flux group (HR = 0.79, 95% CI, 0.54–1.16, *p* = 0.222; HR = 0.58, 95% CI, 0.37–0.91, *p* = 0.019).

**Conclusions:**

High-flux hemodialysis was associated with a lower relative risk of cardiovascular mortality in elderly MHD patients. High-flux hemodialysis did not improve all-cause mortality rate. Differences in urea distribution volume, blood flow, and systemic differences in solute clearance by dialyzers were not further analyzed, which are the limitations of this study.

## Introduction

Maintenance hemodialysis (MHD) is the main renal replacement therapy used for patients with end-stage renal disease (ESRD) [1]. With the development of blood purification techniques, the survival of MHD patients has improved, but their mortality rate is still higher than that of the general population [2]. In China, approximately 750,000 patients with end-stage renal disease were treated with hemodialysis by 2021. The prevalence of ESRD is projected to reach 1505 patients per million population by 2025 [3]. In recent years, the number of elderly patients undergoing hemodialysis has grown rapidly due to the growth of the elderly population and, in particular, the increase in the number of ESRD patients. Data from the United States show that 40% of end-stage patients were older than 65 years in 2013, and projections for 2030 indicate that this proportion will increase to 55–61% [4]. A German study revealed that 72.2% of dialysis patients were over 60 years old [5]. Most elderly hemodialysis patients suffer from more common cardiovascular disease (CVD), malignancies, frailty, malnutrition, diabetes and poorer quality of life than younger hemodialysis patients [6,7], which leads to adverse outcomes in elderly patients [8,9].

The main hemodialysis modalities for MHD patients are low-flux hemodialysis and high-flux hemodialysis. High-flux membranes have the capacity to remove retention solutes of higher molecular weight than low-flux membranes [10]. Despite the better performance of high-flux membranes in the removal of uremic toxins, the clinical benefit of high-flux hemodialysis remains controversial. There are fewer studies on the effect of membrane flux on survival outcomes in the elderly MHD population. Therefore, the purpose of this study was to determine the effect of membrane flux on survival outcomes in elderly MHD patients.

## Materials and methods

### Research design

A retrospective analysis of 1155 patients who underwent hemodialysis treatment from March 2014 to March 2020 at the General Hospital of Northern Theater Command was performed. The endpoint was all-cause or cardiovascular death, and the follow-up deadline was December 1, 2022.

The inclusion criteria were as follows: >60 years of age, >6 months on dialysis, 4 h of conventional hemodialysis treatment 3 times a week, and complete data available. The exclusion criteria were as follows: renal transplantation or conversion to peritoneal dialysis or transfer to other dialysis centers; missing key information, such as demographic information, endpoint events, and laboratory data; presence of tumors; presence of catheters; and interdialytic weight gain greater than 5% of body weight.

**Figure 1.**
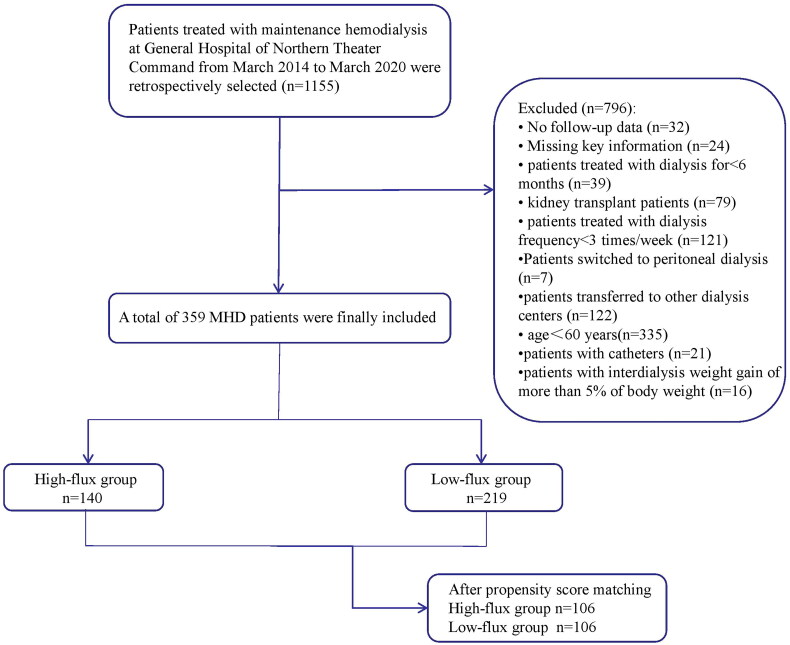
Flow diagram about the number of patients included and excluded from this study.

A total of 359 MHD patients were ultimately included. The study was approved by the Ethics Committee of the General Hospital of Northern Theater Command (approval number Y (2023) 152). As this was a retrospective study, written consent was not needed. More detailed information about the number of patients included and excluded from this study is shown in Figure 1. Patients were categorized into high-flux and low-flux groups based on the dialyzer they used. The dialyzer protocol was not changed for any of the enrolled patients during the entire observation period.

### Dialysis parameters

All patients were treated with a Fresenius 4008B hemodialysis machine, a polysulfone membrane dialyzer, and Tianjin Zhixin dialysate. The concentrations of calcium, potassium, sodium and bicarbonate in the dialysate were 1.5 mmol/L, 2.0 mmol/L, 138 mmol/L and 32.5 mmol/L, respectively. The dialysate flow rate was 500 mL/min, and the blood flow rate was 250-300 mL/min for 4 h three times a week. High-flux dialysis was defined as an ultrafiltration coefficient of ≥ 20 mL/mmHg per hour, and low-flux dialysis was defined as an ultrafiltration coefficient of <20 mL/mmHg per hour [11]. The membrane surface area of the high-flux dialyzers was 1.8 m^2^ (Fresenius Classix FX 80). In low-flux dialysis, the dialyzer membrane has a surface area of 1.6 m^2^ (Fresenius F7). The Kuf values in this study were 16 mL/h/mmHg for low-flux dialyzers and 44 mL/h/mmHg for high-flux dialyzers. K0A was 789 mL/min for low-flux dialyzers and 1263 mL/min for high-flux dialyzers.

### Study data collection

Clinical data were obtained from the hospital electronic medical records and included age, year on dialysis, sex, primary disease, comorbidities, medication data, height, weight, body mass index (BMI) and smoking status.

Laboratory data were obtained from the hospital laboratory management system and were collected for the first time 6 months after patient enrollment, with blood collected intravenously before the start of dialysis treatment. These included serum albumin (Alb), hemoglobin (Hb), calcium (Ca), phosphorus (P), intact parathyroid hormone (iPTH), creatinine (Cr), blood urea nitrogen (BUN), uric acid (UA), β2-microglobulin (β2-mg), Kt/V, and the urea reduction ratio (URR).

### Outcomes

The outcomes were all-cause and cardiovascular mortality. All-cause death was defined as death from any cause. Cardiovascular death was defined as death from sudden cardiac death, myocardial infarction, heart failure, stroke, or other vascular causes [12].

### Statistical analysis

The measurement data are expressed as the mean ± standard deviation (SD). Variables with a nonnormal distribution are expressed as medians with ranges. Normally distributed data were compared with Student’s t test, and nonnormally distributed data were compared using the Kruskal–Wallis (K-W) test. The chi-square test was used for categorical variables. Significantly different demographic data between the two groups were matched by 1:1 propensity score matching (caliper value 0.05). The variables included in the propensity score in this study were age, year on dialysis, hypertension status, diabetes status, CVD status, vitamin D usage, phosphate binder usage, ACEI or ARB usage, and serum ALB, hemoglobin, β2-microglobulin and C-reactive protein levels. In this subcohort of propensity score-matched patients, survival curves were plotted for these patients using the Kaplan–Meier method for the high-flux and low-flux groups, and the log rank test was used for survival comparisons between the two groups. Multivariate Cox proportional hazards analysis was used to assess the effect of membrane flux on survival using three different multivariate models adjusted for hazard ratios (HRs) and 95% confidence intervals (CIs) for all-cause mortality and cardiovascular mortality. Model 1 included demographic information: age, sex and year on dialysis. Model 2 was defined by the addition of comorbidities, including hypertension, diabetes, and CVD; concomitant medications, including vitamin D; phosphate binder usage; and ACEI or ARB usage. Model 3 was defined by the addition of laboratory indicators, including serum ALB, hemoglobin, β2-microglobulin and C-reactive protein concentration, to Model 2. Statistical analyses were performed using SPSS software (version 25.0). Differences were considered statistically significant when *p* < 0.05.

## Results

### Patient baseline characteristics

A total of 140 (39.0%) patients were in the high-flux group, and 219 (61%) patients were in the low-flux group. The baseline characteristics of the patients in the two groups before propensity score matching are shown in Table 1. Compared with those in the low-flux group, patients in the high-flux group had more years of dialysis, higher hemoglobin and serum ALB levels and lower β2-microglobulin levels. There were no significant intergroup differences in blood calcium, phosphorus, creatinine, urea, uric acid C-reactive protein or iPTH between the two groups. Propensity score matching was used to match 106 pairs in the two groups, for a total of 212 individuals.

**Table 1.** Demographic characteristics and laboratory measurements before propensity score matching.

	High-flux group(*n* = 140)	Low-flux group(*n* = 219)	p
Demographic characteristics			
Age (yr)	66.38 ± 5.224	70.19 ± 6.788	0.000
Sex (male; %)	77(55%)	100(45.7%)	0.084
Years on dialysis(yr)	2.97 ± 3.25	2.08 ± 2.89	0.009
Height (cm)	166.71 ± 8.484	163.56 ± 7.946	0.002
Weight (kg)	62.88 ± 12.269	59.71 ± 11.444	0.029
Body mass index (kg/m^2^)	22.51 ± 3.41	22.29 ± 3.73	0.603
Current smokers, *n* (%)	23(16.4%)	39(18.7%)	0.593
Comorbidity			
Diabetes, *n* (%)	50(35.7%)	90(41.1%)	0.333
Hypertension, *n* (%)	109(77.9%)	178(81.3%)	0.508
Cardiovascular disease, *n* (%)	70(50.0%)	149(68.03%)	0.001
Heart failure	27(38.6%)	60(40.3%)	
Cardiac arrhythmia	36(51.4%)	79(53.0%)	
Coronary artery disease	16(22.9%)	45(30.2%)	
Peripheral vascular disease	17(24.3%)	23(15.4%)	
Primary disease			0.018
Chronic glomerulonephritis, *n* (%)	40(28.6%)	37(16.9%)	
Hypertensive renal damage, *n* (%)	29(20.7%)	52(23.7%)	
diabetic nephropathy, *n* (%)	34(24.2%)	80(36.5%)	
Other diseases, *n* (%)	37(26.4%)	50(22.8%)	
Laboratory examination			
Hemoglobin (g/L)	107.26 ± 16.34	104.98 ± 15.76	0.188
Albumin (g/L)	37.45 ± 3.55	36.42 ± 3.64	0.009
Calcium (mmol/L)	2.25 ± 0.21	2.21 ± 0.19	0.093
Phosphate (mmol/L)	1.88 ± 0.46	1.81 ± 0.57	0.243
intact parathyroid hormone (pg/mL)	361.68 ± 382.75	319.58 ± 356.59	0.316
blood urea nitrogen (mmol/L)	24.90 ± 5.29	23.77 ± 5.97	0.078
Creatinine (µmol/L)	932.56 ± 197.38	938.30 ± 211.91	0.804
uric acid (mmol/L)	440.30 ± 75.82	433.88 ± 71.05	0.440
β2-microglobulin (mg/L)	28.76 ± 6.47	31.75 ± 6.98	0.000
C-reactive protein (mg/dL)	5.75 ± 3.49	5.27 ± 2.88	0.172
Kt/V	1.33 ± 0.27	1.39 ± 0.23	0.108
Urea reduction ratio	76.53 ± 5.79	75.87 ± 5.99	0.302
Medication data			
Vitamin D usage (%)	97(69.3%)	131(59.8%)	0.056
Phosphate binder usage (%)	110(78.6%)	156(71.2%)	0.095
ACEI or ARB usage (%)	67(47.9%)	107(48.9%)	0.853

### Survival analysis

**Figure 2.**
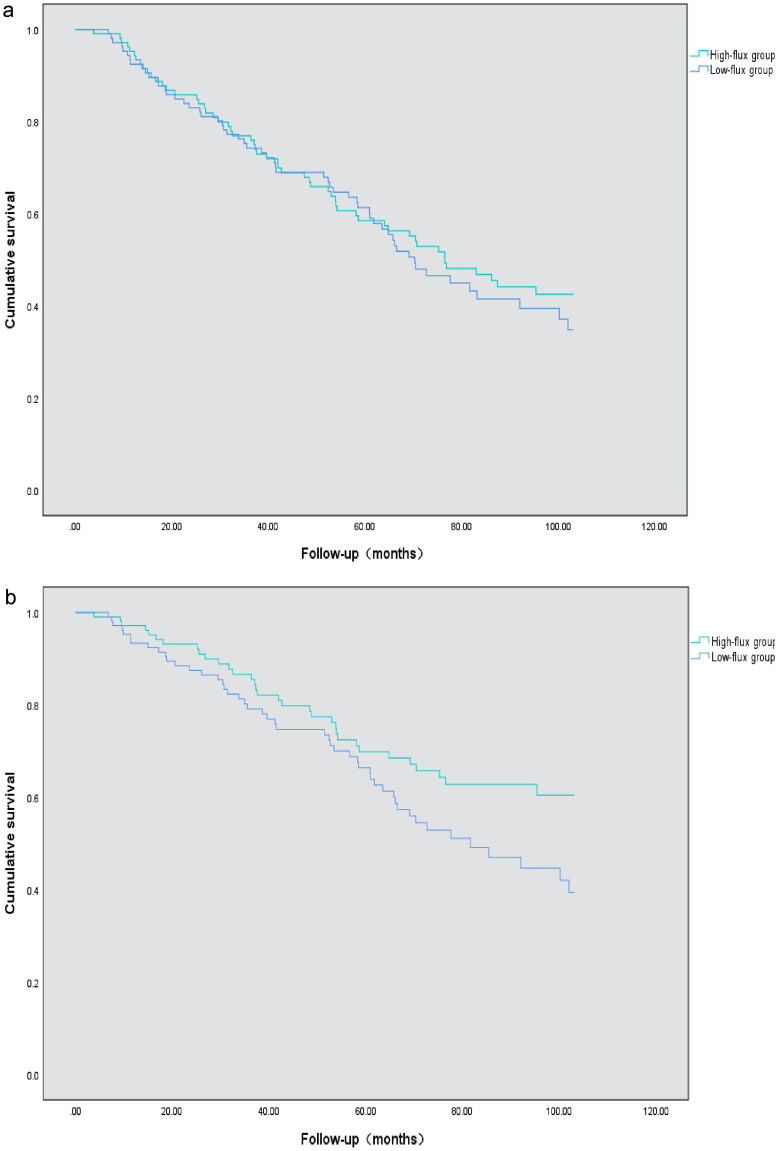
All-cause and cardiovascular survival curves according to low-flux group and high-flux group according to Kaplan–Meier analysis. (a) K–M survival analysis revealed no significant difference in all-cause mortality between the high-flux group and the low-flux group (log-rank test, *p* = 0.559); (b) K–M survival analysis of cardiovascular mortality in the two groups: Patients in the low-flux subgroup had a greater death rate from cardiovascular mortality than did those in the high-flux subgroup (log-rank test, *p* = 0.049).

By the end of follow-up, 112 patients had all-cause death, accounting for 52.8% of the total. There were 82 cardiovascular deaths, accounting for 38.2% of the total. The median follow-up time was 72.68 ± 5.79 months (range 3.8–103.1 months). K–M survival analysis revealed no significant difference in all-cause mortality between the high-flux group and the low-flux group. The log-rank test (*p* = 0.559) was used, as shown in Figure 2a. The cardiovascular mortality rate in the high-flux group was significantly lower than that in the low-flux group according to the log-rank test (*p* < 0.05), as shown in Figure 2b.

### Multivariate Cox proportional hazards analysis

After adjustment for factors such as age, sex and time on dialysis, the HR of all-cause death in the high-flux group versus the low-flux group was 0.84 (95% CI, 0.58 to 1.22; *p* = 0.372 for Model 1). After adjustment for demographic data, comorbidities (hypertension, diabetes, CVD) and concomitant medications, the HR in the high-flux group versus the low-flux group was 0.83 (95% CI, 0.57 to 1.20; *p* = 0.320 for Model 2). After multivariate Cox hazard regression adjustment for demographic data, comorbidities, concomitant medications and laboratory data, the adjusted HR for all-cause death in the high-flux group versus the low-flux group was 0.79 (95% CI, 0.54 to 1.16; *p* = 0.222 for Model 3). High-flux dialysis did not significantly reduce the risk of all-cause mortality. In Model 1, after adjustment for age, sex, and year on dialysis, the HR of cardiovascular death in the high-flux group versus the low-flux group was 0.60 (95% CI, 0.38 to 0.94; *p* = 0.025). In Model 2, after adjusting for demographic data, comorbidities (hypertension, diabetes, CVD) and concomitant medications, we found that the HR of cardiovascular death in the high-flux group versus the low-flux group was 0.59 (95% CI, 0.38 to 0.93; *p* = 0.023). In Model 3, after adjustment for demographic data, comorbidities, concomitant medications and laboratory data, the adjusted HR for cardiovascular death in the high-flux group versus the low-flux group was 0.58 (95% CI, 0.37 to 0.91; *p* = 0.019), and the results showed that high-flux hemodialysis could reduce the risk of cardiovascular death in elderly dialysis patients by 42% (Table 2).

**Table 2.** Multivariate cox regression analysis of variables predicting mortality.

	All-cause mortality		Cardiovascular mortality	
	HR (95% CI)	*p* value	HR (95% CI)	*p* value
Model 1 (demographics)	0.84(0.58–1.22)	0.372	0.60(0.38–0.94)	0.025
Model 2 (Model 1 + comorbidities and concomitant medications)	0.83(0.57–1.20)	0.320	0.59(0.38–0.93)	0.023
Model 3 (Model 2 + Laboratory examination)	0.79(0.54–1.16)	0.222	0.58(0.37–0.91)	0.019

Model 1 was adjusted for age, sex, and year on dialysis.

Model 2 was adjusted for Model 1, and comorbidities and concomitant medications included age, sex, year on dialysis, hypertension status, diabetes status, cardiovascular disease status, vitamin D usage, phosphate binder usage, and ACEI or ARB usage.

Model 3 was adjusted for Model 2 and laboratory examination results and included age, sex, year of dialysis, hypertension status, diabetes status, ­cardiovascular disease status, vitamin D usage, phosphate binder usage, ACEI or ARB usage, serum ALB concentration, hemoglobin, β2-microglobulin concentration and C-reactive protein.

## Discussion

High-flux hemodialysis is widely used in hemodialysis centers and is a hemodialysis modality actively recommended by European guidelines [13]. However, whether the use of high-flux dialyzers improves patient survival outcomes remains controversial. When high-flux hemodialysis was first introduced into our center, most patients did not choose high-flux hemodialysis because of the cost of medical insurance and concerns about the treatment effect. In the face of individual differences in patients, poor cardiac function, malnutrition, and having just undergone hemodialysis, Chinese physicians prefer low-flux dialysis treatment. Moreover, none of the patients changed the mode of dialysis during the observation period. The results of our study demonstrate that high-flux hemodialysis reduces the risk of cardiovascular mortality but not all-cause mortality in elderly patients undergoing maintenance hemodialysis.

The Hemodialysis (HEMO) study group showed no significant effect of membrane flux on survival outcomes, but *post hoc* analyses showed a significant survival benefit in the high-flux group, with a 32% reduction in the relative risk of death in patients with a dialysis vintage > 3.7 years [14]. A prospective study that analyzed the survival of 650 dialysis patients over a 2-year follow-up period supported conclusions similar to those from the *post hoc* analyses of the HEMO study. In patients receiving long-term dialysis therapy, better survival was observed when high-flux dialysis membranes were used [15]. Another well-designed prospective clinical trial is the membrane permeability outcome (MPO) study, which demonstrated that in the overall population, there was no significant difference in survival between patients treated with low-flux or high-flux dialysis. However, in the population with a serum ALB concentration < 4 g/dl, there was a significant improvement in survival in the high-flux subgroup, and a secondary analysis of the MPO study showed that diabetic patients receiving high-flux dialysis had a greater survival rate than those receiving low-flux dialysis, with an adjusted risk reduction of 38% [16]. However, many studies have shown that the use of high-flux membranes is associated with improved outcomes in hemodialysis patients. A study from the database of the German Diabetes and Dialysis (4D) reported the superiority of high-flux dialysis in all-cause and cardiovascular mortality in patients with type 2 diabetes mellitus [17]. In the Current Management Of Secondary Hyperparathyroidism (COSMOS) study, which enrolled 6797 patients, the high-flux group had a 24% lower relative risk reduction in all-cause death and a 39% lower relative risk reduction in cardiovascular death after multivariable adjustment [18]. In a systematic review published by Palmer in 2012, the included studies differed in that there was no statistically significant difference in all-cause mortality between high-flux hemodialysis and low-flux hemodialysis, but high-flux hemodialysis reduced cardiovascular mortality by 15% [19]. However, a recent meta-analysis that included seven randomized clinical studies with a total of 4412 patients revealed that high-flux hemodialysis was superior to low-flux hemodialysis in terms of long-term survival, resulting in a 25% reduction in all-cause mortality and CVD mortality but no significant effect on infection mortality [20]. The meta-analysis has several limitations, as baseline data may have been missing for some of the trials, preventing meta-regression analyses of confounders. Therefore, more studies are needed to confirm the clinical benefits of high-flux hemodialysis.

The dialysis dose is a key factor in the overall management of hemodialysis patients with end-stage renal disease. The K/DOQI guidelines indicate that higher doses of dialysis improve patient outcomes [21]. The National Cooperative Dialysis Study (NCDS) showed that monitoring the clearance of small-molecule solutes is an effective way to quantify the dialysis dose, and urea is a marker of these small solutes [22]. The removal of small solutes is mainly achieved by diffusion, which is affected by factors such as blood flow, dialysate flow, and dialyzer surface area. Studies have shown that the clearance of small molecular solutes increases with increasing blood flow, dialysate flow and membrane surface area [23]. As the membrane area of the dialyzer is different, the mass transfer area coefficient (K0A) is also different, so there is a systematic difference in the removal of small solutes [24]. In addition, the effective clearance of urea in patients is also affected by blood water content, vascular access recirculation, cardiopulmonary recirculation [25]. Many past studies have emphasized that the use of high-flux dialyzers to remove small water-soluble uremic toxins ensures good dialysis quality, affects patient clinical outcomes, and reduces acute and long-term hemodialysis-related complications [26]. There may be differences in Kt/V values under different urea clearance treatments, but Kt/V in this study reflects whether the two groups of patients had adequate dialysis. The results of the HEMO study showed that there was no statistically significant difference between the high-flux group and low-flux group before enrollment or during treatment. In this study, there was no difference in body mass index, only body shape (height and weight) was different, so the contradictory trend of Kt/V between the two groups may be related to the blood flow during the treatment and the differences in height and weight of the patients. The differences resulting from this are also listed as limitations of this study. Observational studies have consistently shown that reduced mortality risk is associated with a higher urea reduction ratio (URR) or Kt/V [27]. However, observational studies mainly provide associations rather than causal relationships and are subject to selection bias and confounding factors. Proof of concept in large randomized clinical trials is therefore needed. The multicenter randomized HEMO study revealed no significant relationship between Kt/V and mortality. High-dose dialysis was not associated with an overall survival benefit compared with standard-dose dialysis (based on K/DOQI guidelines) [28]. This may be because uremic toxins include not only small-molecule plasma solutes but also protein-bound solutes and medium molecules [29]. A few years ago, several studies cast some doubts on the predictive value of Kt/V by demonstrating a J-shaped survival curve, which implied that increasing Kt/V from very low to standard values improved mortality; on the other hand, survival started to decline again for very high Kt/V values [30].

Current dialysis guidelines all recommend ensuring that Kt/V exceeds the prescribed minimum level but do not explicitly consider age or frailty [31]. Increasing age is accompanied by physiological and pathological changes that may alter the patient’s response to uremia and dialysis. Therefore, compared with younger patients, elderly patients tend to have lower serum concentrations of these toxins if the clearance is the same [32]. However, regardless of the concentration of uremic toxin, body water volume (V) has an important effect on Kt/V [33]. The higher Kt/V values are due to lower V and poor nutritional parameters. In elderly individuals, more comorbidities may have a greater adverse impact on quality of life than uremic symptoms; thus, the dialysis dose may not be as important. Higher doses of dialysis may be more difficult to reach and less tolerable in elderly patients. Dialysis prescriptions for elderly patients should be individualized, and multiple factors should be considered comprehensively [32]. In a study of solute clearance during high-flux versus low-flux dialysis, there was no statistically significant difference in the urea reduction ratio (URR) between the two groups, which is consistent with the results of the present study [34]. A study of the relationship between hemodialysis adequacy and quality of life in older adults revealed that for a urea reduction ratio (URR) ranging from 64.0% to 88.9% in elderly patients undergoing hemodialysis, improving hemodialysis adequacy beyond these levels may not be necessary to maintain the quality of life of elderly patients [35].

Hemoglobin levels are correlated with cardiovascular events and mortality in many observational hemodialysis studies [36]. The DOPPS included a study on anemia management that showed that low hemoglobin levels were associated with a greater risk of death [37]. Elderly dialysis patients have low hemoglobin levels [38]. In elderly individuals, quality of life is also likely to improve when hemoglobin levels are elevated. Second, cardiac function can also improve strikingly when slightly below-normal hemoglobin concentrations are elevated to the mid-normal range [39]. High-flux dialysis may improve patient hemoglobin levels through improved clearance of moderate- and high-molecular-weight toxins [40]. The average weekly dose of human recombinant erythropoietin required to maintain target hemoglobin levels on high-flux dialysis is lower than the average dose needed in patients on low-flow dialysis [41]. In addition, a randomized prospective study revealed that patients on high-flux dialysis had a significantly greater rate of goal Hb attainment than did those in the low-flux group, and high-flux dialysis was beneficial for maintaining stable hemoglobin levels [42].

CVD is known to be the leading cause of death in ESRD patients, accounting for nearly 50% of deaths in the hemodialysis population [43]. ESRD patients may have progressive congestive heart failure and cardiomyopathy due to coronary artery disease, increased left ventricular wall thickness, increased left ventricular mass, and impaired systolic and diastolic function caused by risk factors such as hypertension, uremic toxins, and anemia [44]. Older HD patients are more likely to have a combination of atherosclerotic lesions and cardiac insufficiency and are therefore considered at high risk for CVD [45]. These conditions reduce patients’ tolerance to HD ultrafiltration and increase the risk of hypotension during HD. Hypotension usually causes symptoms and may result in early termination of dialysis. Repeated episodes may result in damage to the heart and brain [46]. Moreover, uremic toxins have been shown to have direct effects on cardiac structure and function, leading to interstitial fibrosis, reduced cardiac contractility, and thus impaired cardiac function [47]. High-flux dialysis has been shown to provide significant clearance of intermediate molecular toxins and to reduce β2-microglobulin levels [48]. It has been shown that impaired diastolic function predisposes patients to dialysis hypotension, which can cause endocardial ischemia and malignant arrhythmias. A double-blind, single-crossover study of the effects of high-flux hemodialysis on left ventricular function in patients with ESRD revealed an improvement in systolic function during high-flux therapy [44]. Elderly patients have vascular stiffness and reduced vascular regulatory function due to abnormal vascular wall structure, decreased elastin, and reduced central arterial compliance. This leads to reduced tolerance to ultrafiltration and susceptibility to hypotension during dialysis, and recurrent hypotension during dialysis significantly increases cardiovascular mortality [5]. A review and meta-analysis of 5 studies comprising a total of 2612 patients revealed that the use of high-flux membranes was associated with decreased cardiovascular mortality [19], which is consistent with the results of our study.

The strength of this study is the application of propensity score matching (PSM) to reduce bias, but this study also has several limitations. First, we performed propensity score matching or multivariate Cox proportional hazards regression models using variables after the initiation of high- or low-flux therapy, which is a major limitation of our study. Second, there were differences in height and weight between the two groups in this study. Therefore, there were also differences in the urea distribution volume. We did not analyze height, weight, blood flow, or weight gain further. Finally, systematic differences in urea clearance between dialyzers were not analyzed in this study. In this study, only the same treatment time and dialysate flow rate were guaranteed, but the differences in membrane area and blood flow also affect the removal of small solutes. Therefore, in this study, there was a systematic difference of 10% in the theoretical small solute clearance between the two groups. This was a retrospective study, and bias may still have affected the results after propensity score matching. Patients in the high-flux group had been previously treated with low-flux agents before this study, and the effect of prior HD on clinical outcomes was not analyzed or considered.

## Conclusion

This study showed that high-flux hemodialysis did not reduce the risk of all-cause mortality but did reduce the risk of cardiovascular mortality in elderly MHD patients. These results suggest that high-flux hemodialysis should be recommended for the management of elderly MHD patients. However, better-designed and more comprehensive trials are needed.

## Data Availability

The datasets used in this study are available from the corresponding author upon reasonable request.
